# Reply to the Letter to the Editor Entitled “Optimal risk stratification and therapeutic strategy for acute myocardial infarction”

**DOI:** 10.1002/clc.23646

**Published:** 2021-05-22

**Authors:** Sheng‐ji Wang, Hai‐ying Zhao, Xiao‐ting Fan

**Affiliations:** ^1^ Department of Emergency Linyi People's Hospital Affiliated to Shandong University Linyi Shandong China; ^2^ Department of Neurosurgery ICU Linyi People's Hospital Affiliated to Shandong University Linyi Shandong China

To the Editor,

We appreciate the comments of Imamura^1^ about our article entitled “Development of an optimized risk score to predict short‐term death among acute myocardial infarction patients in rural China.”^2^ We would like to state that this was a retrospective single‐center study, which aimed to establish an optimized risk score to predict short‐term (6‐month) death among rural AMI patients from China.

The GRACE risk score, one of risk prediction tools have been developed to assess short‐term or long‐term death risk for acute coronary syndrome (ACS) patients, remains the most popular and validated model in rural China. Since our scoring method shares a similar study object and primary endpoint event as the GRACE risk model, we used the latter to contrast and validate the predictive power of the optimized risk score model herein described. We will compare the optimized risk score model with other scorings (such as TIMI risk score, PURSUIT risk score, or NCDR‐ACTION registry) in future to enhance its clinical implications.

Several parameters such as blood glucose and pulmonary artery systolic pressure included in their risk score are modifiable.^1^ The investigation of whether mortality would improve when these parameters are ameliorated will be the subject of our further study.

Teruhiko Imamura stated that the prognostic impact of several parameters such as blood glucose might be “U‐shape” instead of linear, but we could not agree with it. Hyperglycemia is very common in AMI patients, while hypoglycemia is rare.^3^ Previous studies have shown that blood glucose levels are commonly elevated in early AMI and represent an independent risk factor for increased in‐hospital or short‐term mortality.^4^, ^5^ Similarly, pulmonary hypertension is frequently observed following AMI,^6^ and elevated PASP after AMI is often associated with poor prognosis.^7^

Whether the cubic spline model is more suitable to represent the accurate association between these parameters and mortality should be verified. We have proved that the optimized risk score is superior to the GRACE risk model, and we expect to provide clinicians with a simple and useful tool to accurately assess short‐term death risk and to select appropriate treatment and level of care for AMI patients.
